# MHC Class II Tetramers Made from Isolated Recombinant α and β Chains Refolded with Affinity-Tagged Peptides

**DOI:** 10.1371/journal.pone.0073648

**Published:** 2013-09-02

**Authors:** Peter Braendstrup, Sune Justesen, Thomas Østerbye, Lise Lotte Bruun Nielsen, Roberto Mallone, Lars Vindeløv, Anette Stryhn, Søren Buus

**Affiliations:** 1 Laboratory of Experimental Immunology, University of Copenhagen, Copenhagen, Denmark; 2 Allogeneic Hematopoietic Cell Transplantation Laboratory, Department of Hematology, Rigshospitalet, Copenhagen, Denmark; 3 Institut National de la Santé et de la Recherche Médicale, Unité 1016, Cochin Institute, DeAR Lab Avenir, Paris, France; University of Palermo, Italy

## Abstract

Targeting CD4+ T cells through their unique antigen-specific, MHC class II-restricted T cell receptor makes MHC class II tetramers an attractive strategy to identify, validate and manipulate these cells at the single cell level. Currently, generating class II tetramers is a specialized undertaking effectively limiting their use and emphasizing the need for improved methods of production. Using class II chains expressed individually in *E. coli* as versatile recombinant reagents, we have previously generated peptide-MHC class II monomers, but failed to generate functional class II tetramers. Adding a monomer purification principle based upon affinity-tagged peptides, we here provide a robust method to produce class II tetramers and demonstrate staining of antigen-specific CD4+ T cells. We also provide evidence that both MHC class II and T cell receptor molecules largely accept affinity-tagged peptides. As a general approach to class II tetramer generation, this method should support rational CD4+ T cell epitope discovery as well as enable specific monitoring and manipulation of CD4+ T cell responses.

## Introduction

CD4+ T lymphocytes are arguably the most important cells of the adaptive immune system. Their primary effector function is to control a range of immune cells (e.g. B cells, CD8+ T cells, and macrophages), which allow them to orchestrate and regulate immune responses against any real or perceived threat (recently reviewed by Paul and coworkers [1]). Thus, they are vitally important for the generation of appropriate and effective immune responses (including immunological memory) against a large variety of pathogenic microorganisms and tumors; and they are also implicated in the inadvertent generation of immune responses against autoantigens, allergens, transplants and pharmaceutical proteins. The underlying specificity of a CD4+ T lymphocyte is exerted through a highly diverse and clonally distributed set of T cell receptors (TcR). Each CD4+ T cell clone expresses a unique TcR variant enabling this particular clone to survey a part of the universe of antigens. Possessing a large number of different clones, the CD4+ T lymphocyte arm of the immune system is in principle capable of covering the entire universe of protein antigens. TcR-driven cellular selection processes activate and expand some clones, while other clones are inactivated and/or deleted, thereby determining the specificities actually possessed by the T cell repertoire of a given individual. Ideally, these selection processes should establish and maintain immunity against pathogens, and at the same time avoid autoimmunity.

It follows that a thorough understanding of how the specific immune system works, and how it can be manipulated and exploited, would benefit tremendously from efficient, reliable and highly discriminatory methods to identify, enumerate, monitor and characterize individual CD4+ T cell specificities. Unfortunately, this is an extraordinary experimental challenge since each clonally distributed TcR is expressed by a very small fraction of the total number of CD4+ T cells. Identification and discrimination between different CD4+ T cell specificities at the level of individual T cells requires that the TcR itself be targeted. To this end, one would have to use the specific ligand recognized by the TcR as the targeting principle, but such an approach is complicated by serious immunological and technical problems. The relevant TcR ligand is in itself a complex structure consisting of a specific antigen-derived peptide bound to a major histocompatibility class II molecule (generically called MHC; in humans denoted human leucocyte antigens (HLA)), and neither of these components may be known to the experimentalist; actually, a frequent purpose of addressing a CD4+ T cell response is to establish the identity of the peptide antigen and its MHC class II restriction element. Once the relevant peptide-MHC class II components are known, two serious challenges remain: 1) how to generate the corresponding peptide-MHC class II complexes, and 2) how to make them bind to their target TcR in a way that overcome the very unstable nature of this interaction [2], thereby allowing detection of specific CD4+ T cells.

Almost two decades ago, Altman and coworkers provided a general solution to the inherent problem of the unstable nature of the interaction between T cell receptors and cognate peptide-MHC complexes [3], [4]. Using multimeric peptide-MHC complexes to increase the stability of productive interactions, they could detect antigen-specific, MHC-restricted T cells at the single cell level. The preferred approach to multimerize peptide-MHC complexes was – and still is – to introduce a biotin tag into a monomer peptide-MHC complex and then use the ability of streptavidin (SA) to bind biotinylated peptide-MHC monomers with high affinity and assemble them into peptide-MHC tetramers (hence any multimeric MHC complex is denoted “MHC tetramers”). Originally, they demonstrated that peptide-MHC class II tetramers could label specific CD4+ T lymphocytes [3] and that peptide-MHC class I tetramers could label specific CD8+ T lymphocytes [4]. Since then, MHC class I multimers have rapidly transformed the field of cellular immunology, effectively becoming the golden standard for direct enumeration, analysis and manipulation of CD8+ T cells [5]. In contrast, generating and using MHC class II multimers to characterize CD4+ T cells have shown much slower progress and have been less successful [6]. Studies have suggested that fundamental differences between CD4+ and CD8+ T cells exist that may explain the reduced ability to stain CD4+ T cells with tetramers [6], [7], [8]. In peripheral blood, the frequencies of antigen-specific CD4+ T cells are lower: in the 10^−3^ to 10^−4^ range for boosted cells, 10^−4^ to 10^−5^ for resting memory cells, and 10^−5^ to 10^−7^ for naïve cells [8], [9]. Obtaining measurable numbers of CD4+ T cells often requires higher frequencies; something that can be obtained by specific expansion through *in vitro* culture [10], which is likely to alter the functional state of the cells; or by specific capture-based purification from a large sample of cells obtained *ex vivo* [9], [11], [12]. Another difference involves the accessory molecules, where the interaction between a TcR and its cognate peptide-MHC complex is stabilized by CD8, but not by CD4 [13], [14], [15]. Finally, studies have shown that the affinity of TcR interacting with peptide-MHC class II complexes is significantly lower than the affinity of TcR interacting with peptide-MHC class I complexes [16]. This may be a particularly important limitation in the ability to detect anti-self and anti-tumor CD4+ T cell populations [7], [16]. In reality, only antigen-specific CD4+ T cells of the highest frequency, avidity and/or affinity may be detectable [17].

Other obstacles to progress in the development of an efficient MCH class II tetramer technology derive from challenges in design and/or production of recombinant MHC reagents. Over the past decades we have developed versatile, high-yield, recombinant *E. coli* expression systems generating highly active, biotinylated MHC class I [18] and II [19] molecules and accompanying high throughput peptide-binding assays [19], [20]. For MHC class I, we have also developed a “one-pot, mix-and-read” tetramer approach where the consecutive admixture of MHC class I, peptide and SA (i.e. without any intervening steps) leads to the generation of MHC class I tetramers [21]. Unfortunately, a similar “one-pot, mix-and-read” approach has so far failed for MHC class II (unpublished observations). Here, we report an alternative approach to generate peptide-MHC class II tetramers. Recombinant MHC class II α and β chains (the latter being biotinylated *in vivo*) were refolded *in vitro* in the presence of synthetic peptides that had been extended by a hexa-histidine (H_6_) sequence. The resulting peptide-MHC class II complexes (in this context denoted “monomers”) could readily be purified and concentrated by immobilized metal affinity chromatography (IMAC), and could then be tetramerized using fluorochrome-labeled SA. Here, we demonstrate that these MHC class II tetramers can be used to stain and purify antigen-specific, MHC class II-restricted CD4+ T lymphocytes. We propose that this is a fast, general, and efficient method to produce peptide-MHC class II tetramers.

## Materials and Methods

### HLA Class II Constructs

The construction of HLA class II genes has been described previously [19]. Briefly, HLA-DR class II α chains were truncated at position 191 and C-terminally fused to a FOS leucine zipper segment. HLA-DR class II β chains were truncated at position 190 and C-terminally fused to a JUN leucine zipper segment and a biotinylation sequence peptide (BSP). HLA genes were codon-optimized for *E. coli* expression, generated synthetically by GenScript (Piscataway, NJ, USA), cloned into the pET28a+ (kanamycin resistance, IPTG induction, Novagen®, EMD Millipore Billerica, MA) *E. coli* expression vector, and transformed into DH5α *E. coli* cells using standard molecular biology techniques. The intended DNA sequences were verified by DNA sequencing (ABI3100, Perkin Elmer). For protein production purposes, plasmids were purified and transformed into BL21(DE3) *E. coli* cells. To allow for *in vivo* biotinylation of the HLA class II β chains, appropriately transformed BL21(DE3) cells were co-transformed with a pASYC (chloramphenicol resistance, IPTG inducible) vector containing a gene encoding the BirA biotinylation holoenzyme. Clones of transformed BL21(DE3) cells that expressed the intended recombinant product upon induction with IPTG, as determined by SDS-PAGE, were identified and stored at −80°C.

#### Expression of MHC class II α and β chain proteins in E. coli inclusion bodies and purification of denatured MHC class II α and β chains

As previously described, transformed BL21(DE3) cells were grown in a lab-scale fermentor, and MHC class II α or β chains were expressed using IPTG induction [22]. Briefly, cells were expanded overnight and used to seed a 2.5 L Labfors® fermentor. Cells were grown at 37°C to an OD of 25. The temperature was then raised to 42°C and IPTG added to a concentration of 1 mM. For in vivo biotinylation of β chains, 0.5 mM d-Biotin was added at the time of induction. After 3 hours, cells were collected and processed at 2.3 kBar in a cell disrupter (basic Z, Constant Systems Ltd Daventry, UK). Using centrifugation (Sorvall RC6, 17,000 g, 30 min, 4°C), the inclusion body pellet was washed twice in 0.5% NP40, 0.1% DOC in PBS. The washed pellet was dissolved overnight in 200 ml 8 M Urea, 25 mM Tris, pH 8, and any remaining DNA was precipitated with streptomycin sulphate (10 g/L). After centrifugation, the denatured protein solution was applied to an 800 ml Q Sepharose Fast Flow column. The column was washed with 8 M Urea, 25 mM Tris, pH 8 (Buffer A), and eluted in buffer A containing 1 M NaCl (Buffer B) using a two-step gradient (a 0–30% B gradient over 3 column volumes (CV) followed by 100% B over 1 CV). Fractions containing proteins of interest, as determined by SDS-PAGE, were pooled and concentrated to 100 ml using tangential ultrafiltration (10 kDa cut-off, Vivaflow 200, Vivascience AG, Göttingen, Germany). The concentrate was applied to a 3.5 L Superdex 200 PG gel filtration column (GE Healthcare) and eluted in 8 M Urea, 25 mM Tris, 150 mM NaCl, pH 8. Fractions containing denatured HLA class II α or β chains were pooled, diluted to 10 µM and stored at −80°C.

#### Peptide-MHC class II binding measurements

Aiming at the highest signal-to-noise ratio of peptide binding, optimal concentrations of the recombinant HLA class II α and β chains were identified for each individual α-β chain combination in pilot experiments as previously described [19]. Measurements of peptide-MHC class II binding were carried out as previously described [19]. Briefly, peptides were dissolved in DMSO to 0.4 mM and further diluted into refolding buffer (RFB; 25% Glycerol, 50 mM Tris/Citrate, pH 7 containing 0.01% Pluriol F68 and protease inhibitors 5 µM pepstatin A, 460 µM PMSF, 9 µM TLCK and 30 µM TPCK) to a concentration of 40 µM, and then 5-fold serially diluted in refolding buffer; 15 µl of each titration were distributed in Optiplates (PerkinElmer). Subsequently, 15 µl of an MHC class II solution containing urea denatured α and β chains were added (prediluted in RFB to the optimal concentrations of the two chains) and the reaction mixtures were incubated for 48 h at 18°C. The resulting peptide-MHC class II complex formation was measured by a luminescence oxygen channeling immunoassay (LOCI, commercialized as AlphaScreen by Perkin Elmer (Waltham, MA, USA) as previously described [19]. Briefly, 15 µl PBS/0,01% Tween 20 containing 225 ng “donor” beads coated with SA and 225 ng “acceptor” beads coated with the pan-specific HLA-DR monoclonal antibody L243 were added to each well and incubated overnight at 18°C. The “acceptor” beads had been coupled according to the manufacturer’s instructions. Finally, the reactions were read in an Envision reader (PerkinElmer) and the concentrations of peptide-MHC class II monomer were determined by curve fitting to a standard as previously described [19].

#### Refolding and purification of peptide MHC class II complexes using H6-tagged peptides

MHC class II α and β chains (1 ml containing 10 nmoles of each) were mixed and diluted drop-wise and under stirring into 50 ml RBF containing 2 µM (100 nmoles) of a H_6_-tagged peptide of interest. After 48 h incubation at 18°C, the refolding mixture was loaded onto a 6 ml Ni^2+^ Chelating Sepharose Fast Flow (Iminodiacetic acid (IDA) Sepharose, GE Healthcare). The column was washed with PBS until the UV280 signal reached baseline followed by a two-segment step gradient (PBS supplemented with 50 mM Imidazole for 2 CV, and PBS supplemented with 250 mM Imidazole for 2 CV). Fractions were collected and analyzed by reducing SDS-PAGE and by peptide-HLA class II binding assay (see below). Fractions from the 100% elution step containing monomeric peptide-MHC class II complexes were pooled, concentrated on Vivaspin spin filters (10 kD cut off), protein concentration was determined by BCA assay (Pierce®, ThermoScientific, Rockford, IL, USA).

#### Preparation of MHC class II tetramers

MHC class II tetramers were generated by adding SA-phycoerythrin (PE) or SA-allophycocyanin (APC) at an SA:MHC class II ratio of 1∶4. To ensure maximum saturation of the SA with peptide-MHC class II monomers (i.e. that tetramers are formed), SA was sequentially added in six equally divided amounts in 10 min intervals. The resulting MCH class II tetramers were stored as stocks in PBS at 4°C.

### Peptides

The following peptides were used for in vitro stimulation of T cells and for MHC class II tetramer generation: the multiple HLA-DR (including HLA-DRB1*01∶01, HLA-DRB1*04∶01, and HLA-DRB5*01∶01) restricted Influenza A peptide HA_307–318_ (YKYVKQNTLKLAT) [23] (and this publication), the HLA-DRB1*04∶01-restricted Influenza A peptide MP_60–73_ (LGFVFTLTVPSERG) [24], and the HLA-DRB5*01∶01-restricted cytomegalovirus (CMV) peptide IE1_211–225_ (NIEFFTKNSAFPKTT) (this publication). Peptides used as negative control included the HLA Class II-associated invariant chain peptide (CLIP) [25] (LPKPPKPVSKMRMATPLLMQALPMY) and the multiple HLA-DR (including HLA-DRB1*04∶01) restricted Hepatitis C (Hep C) peptide NS3_218–235_ [26], [27] (YAAQGYKVLVLNPSVAAT). All peptides, with and without an additional H_6_ sequence at the C-terminus, were synthesized by Schafer-N, Copenhagen, Denmark.

#### Collection of blood samples and isolation of PBMCs

The study was approved at the National University Hospital of Copenhagen by “The Committees on Biomedical Research Ethics of the Capital Region” (Danish: “De Videnskabsetiske Komitéer for Region Hovedstaden”) (RH-3-CT5604). Informed written consent was obtained from blood donors (age range: 35–65 years). Buffy coats were drawn at The Blood Bank at Rigshospitalet, Copenhagen, Denmark. PBMCs were isolated from the buffy coats by density gradient centrifugation using Lymphoprep (Nycomed Pharma AS, Oslo, Norway) and vials of 20×10^6^ cells were cryopreserved at −150°C in fetal calf serum containing 10% DMSO. Genomic DNA isolated from PBMCs (Qiagen) was subjected to high-resolution sequence-based typing of the HLA-A/B/C and HLA-DR/DQ/DP loci (Genome Diagnostics, Utrecht, the Netherlands).

#### Cell culture

PBMCs were thawed and cultured (10^7^ cells/ml) in X-vivo 15 (Lonza Ag, Cologne, Germany) supplemented with 5% heat inactivated human AB serum (complete media) at 37°C, 5% CO_2_ in humidified air and each peptide was added to a final concentration of 1 µM. After 18–24 h incubation, the cells were washed and resuspended (5×10^6^ cells/cm^2^/ml) in complete media supplemented with IL-2 (final concentration 50 U/ml). From day 5, half of the media was changed every 2^nd^ day and fresh IL-2 was added. IL-15 (final concentration 15 ng/ml) was added from day 6. Cells were harvested on day 12–14.

#### Intracellular cytokine secretion assay and FACS analysis

T cells were analyzed in a standard 4 h intracellular cytokine secretion assay (ICS). Briefly, cells were incubated in complete media with or without 1 µM peptide for 4 h at 37°C in a 5% CO_2_ humidified air atmosphere. Brefeldin A (SigmaAldrich) was added to a final concentration of 10 µg/ml after 1 h of incubation. The cells were subsequently stained according to the ‘FastImmune’ protocol (Pharmingen, San Diego, CA, USA) with anti-CD3, anti-CD4, anti-CD8, anti-CD69, anti- IFN-γ, and anti-TNF-α antibodies (Biolegend and BD Biosciences), detected by a FACS LSRII (BD Biosciences) and analyzed using DIVA II software (BD Biosciences).

#### MHC class II tetramer staining

In vitro stimulated PBMCs were washed in PBS with 1% AB-serum, pelleted and re-suspended in 100 µl of PBS containing 1% AB-serum and 12.5 nM (final concentration) PE- and/or APC-conjugated MHC class II tetramers. The PBMCs were incubated for 1 h at 37°C, 5% CO_2_ in humidified air, washed and subsequently stained with anti-CD3, anti-CD4 antibodies (Biolegend) at 4°C for 30 minutes. After two washes and 1% formaldehyde fixation the cells were analyzed on a FACS LSRII using Diva II software.

## Results

### Binding to HLA Class II Molecule of C-terminally H_6_-tagged Peptides

As an approach to the preparation of peptide-HLA class II monomers, the antigenic peptides were synthetically extended with a C-terminal H_6_ sequence, which subsequently allowed functional, monomeric peptide-HLA complexes to be concentrated and purified by IMAC chromatography. This raises the question whether HLA class II in general will accept this kind of extension. We have previously systematically examined the effect of extending a core class II binding sequence with various oligopeptide sequences of different conformational propensity, and found, that they retained class II binding activity; however, the effect ranged from a 10-fold increase to a 100-fold decrease in affinity [28]. To evaluate the effect of a C-terminal H_6_-extension strategy, we used a LOCI-driven biochemical peptide-HLA-class II binding assay to examine the affinity of four peptides and their H_6_–extended variants to three HLA class II molecules. This selection included ten productive (i.e. binding) peptide-HLA class II combinations. The extended peptides retained their binding status in all ten cases; the effect ranged from a two-fold increase to a three-fold decrease in binding affinity, and a highly significant correlation between bindings of H_6_ vs. non-H_6_ extended peptides was observed (Table 1). Also included were two non-binding peptides, and in both cases the extended peptide remained a non-binder (data not shown). Although the data is limited at this point, it would suggest that HLA class II molecules accept that peptide binders are extended C-terminally with a H_6_ sequence.

**Table 1:** Peptide-HLA class II affinity measurements (in nM) of H_6_- vs. non-H_6_-tagged peptides with four different HLA class II molecules.

Peptide		HLA-DRB1*01∶01		HLA-DRB1*04∶01		HLA-DRB1*15∶01		HLA-DRB5*01∶01	
		K_D_ (nM)		K_D_ (nM)		K_D_ (nM)		K_D_ (nM)	
Peptide Origin	Peptide Sequence	− tag	+ tag	− tag	+ tag	− tag	+ tag	− tag	+ tag
Influenza A MP_60–72_	LGFVFTLTVPSERG	17	40	29	128	270	282	7	13
CMV IE1_211–225_	NIEFFTKNSAFPKTT	83	42	NB	NB	424	127	8	8
Influenza A HA_306–318_	YKYVKQNTLKLAT	9	19	185	394	25	345	4	17
Hep C NS3_218–235_	YAAQGYKVLVLNPSVAAT	3	5	24	42	2	2	68	37

NB, non-binding peptide.

### Generation of Peptide-HLA Class II Monomers

**Figure 1:**
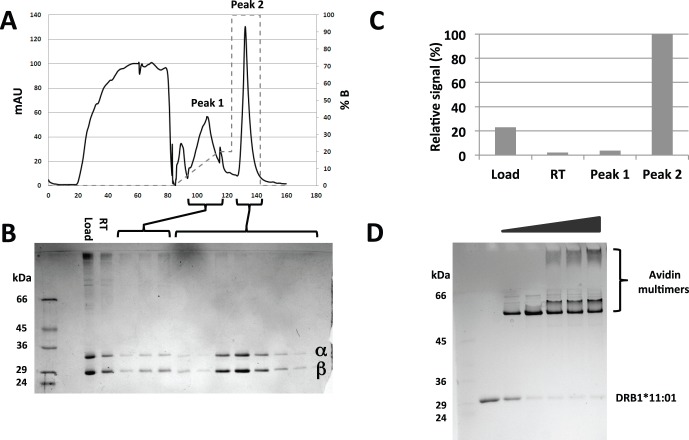
Purification of H_6_-tagged MHC class II complexes. A) Preparative purification of DRB1*04∶01 refolded with Hep C-NS3_218–235(H6)_ (YAAQGYKVLVLNPSVAATHHHHHH) on IMAC column, a shallow gradient of 20% 8 M Urea, 25 mM Tris, pH8, and 1 M NaCl (buffer B) elutes peak 1 whereas peak 2 elutes by raising the concentration of buffer B to 100%. B) SDS-PAGE analysis of eluted fractions, the αchain travels as the upper band. C) Functional LOCI assay on load, run through, and peak fractions. Notice the absence of peptide-loaded MHC class II molecules in the run through (RT) and in peak 1. D) Assessing the level of biotinylation of a DRB1*11∶01 chain by incubation with increasing amounts of avidin followed by SDS-PAGE analysis.

As previously described (see materials and methods), HLA class II α and β chains were individually expressed in *E. coli*, extracted from inclusion bodies, purified by standard chromatography methods, and stored until use as highly active, pre-oxidized, denatured molecules in 8 M Urea, 25 mM Tris, 150 mM NaCl, pH 8 at −80°C. At the time of peptide-HLA class II monomer formation, equimolar amounts of the relevant α and β chains were mixed and diluted drop-wise into a refolding buffer containing excess H_6_-tagged peptide of interest (e.g. final concentrations of 400 nM of each HLA chain, and 2 µM peptide), and incubated at 18°C for 48 h. The resulting peptide-HLA class II monomers were captured and purified by IMAC chromatography. Since β chains on their own bind moderately to Ni^2+^-IDA-agarose, a two-step gradient was used to allow separation of relevant monomers from irrelevant reaction components (i.e. isolated α and β chains, and α-β chain pairs that have dimerized without peptide). An example is shown for HLA-DRA1*01∶01/HLA-DRB1*04∶01 (henceforth DRB1*04∶01) associated with a H_6_-tagged peptide, HA_307–318(H6)_, where the IMAC chromatography was analyzed by absorbance (OD280, Figure 1A), by SDS-PAGE (Figure 1B), and by L243 staining of selected fractions (Figure 1C). L243 is a conformation-dependent monoclonal anti-HLA-DR antibody specific for a monomorphic epitope on the α chain of a properly folded HLA-DR molecule [29], [30] that is strongly supported by peptide binding [19], [31]. For the molecules found in the early eluting peak 1 (Figure 1A), the presence of α and β chains in a 1∶1 stoichiometry (Figure 1B), but lack of L243 staining (Figure 1C), demonstrated that the two chains had associated, but failed to fold properly. A reasonable explanation of this apparent discrepancy is that the α and β chains were kept together by the leucine zipper in a soluble, but inactive, peptide-free form, and that this complex was bound to the Ni^2+^-IDA-matrix through the weak β chain interaction. In contrast, for the molecules found in the later eluting peak 2 (Figure 1A), the presence of α and β chains in a 1∶1 stoichiometry (Figure 1B), and of L243 staining (Figure 1C) demonstrated the presence of properly folded monomer. The elution profile of Peak 2, being clearly separable and delayed compared to Peak 1, strongly supported that these monomers carried the intended H_6_-tagged peptide, as later confirmed by peptide-specific tetramer staining of T cells (see below). The efficiency of the purification method is underlined by the absence of functional complexes in the run-through (Figure 1C). We conclude that this is a simple and robust method to purify peptide-HLA class II monomers and we have routinely obtained monomers in mg quantities.

To support SA-mediated tetramerization, these monomers also needed to be biotinylated. We have previously demonstrated that co-expression of the BirA holoenzyme allows very efficient *in vivo* biotinylation in *E. coli* of proteins of interest that have been fused to a BSP [19], [21]. Thus, a BSP-sequence was inserted after the leucine zipper that had been fused to the β chain. Ideally, the resulting β chain preparations should be fully biotinylated. The degree of biotinylation was determined for all recombinant β chain preparations. Using an increasing amount of SA to saturate the biotinylated β chain preparation in question, the fraction of non-biotinylated β chain could readily be detected in a gel-shift SDS-PAGE. An example of this kind of analysis is shown for a preparation of HLA-DRB1*11∶01 (Figure 1D) demonstrating a virtually complete biotinylation; routinely, biotinylation levels above 95% were achieved.

### Generation of Peptide-HLA Class II Tetramers

The fractions of peak 2 were pooled and the concentration of monomers was determined using a previously described quantitative high-throughput binding assay [19] (for details, see Materials and Methods). The molar amounts of SA needed to obtain fully monomer-saturated SA molecules (i.e. peptide-HLA class II tetramers) were calculated assuming a SA:monomer stoichiometry of 1∶4. To ensure that the vast majority of SA molecules would be saturated, SA was added gradually to monomers under continuous stirring. We routinely generate peptide-HLA class II tetramers in 4 nmol (200 µg) quantities (or corresponding to 200 staining tests). In this study, we used PE or APC conjugated SA to enable flow cytometric detection of antigen-specific CD4+ T cells, but we have also successfully performed HLA class II tetramer staining with other commercially available fluorochromes (e.g. Brilliant Violet 421).

### HLA Class II Tetramer Staining Depends on Temperature, Time, and Intact Metabolism

**Figure 2:**
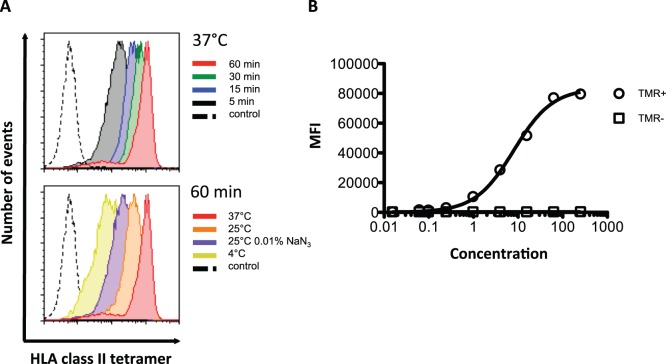
Optimization of time and temperature parameters and concentration of MHC class II tetramer. A) The staining optimization was performed on an HA_307–318_-specific CD4+ T cell line (approximately 100% antigen-specific) with HLA-DRB1*04∶01-HA_307–318(H6)_ and HLA-DRB1*04∶01-Hep C NS3_218–235(H6)_ PE-conjugated tetramers at a concentration of 12.5 nM. Top histograms illustrate staining with HLA-DRB1*04∶01-HA_307–318(H6)_ at 37°C with varying time intervals (black: 5 min; blue: 15 min; green: 30 min; red: 60 min). The dotted black line depicts cells stained with DRB1*04∶01-Hep C NS3_218–235(H6)_ for 60 min at 37°C (control). Bottom histograms illustrate staining with DRB1*04∶01-HA_307–318(H6)_ for 60 min with varying temperature and conditions (lime: 4°C; purple: 25°C and exposure to 0.01% NaN_3_; orange, 25°C; red: 37°C). The dotted black line illustrates cells stained with DRB1*04∶01-Hep C NS3_218–235(H6)_ for 60 min at 37°C (control). B**)** Titration of PE-conjugated HLA-DRB1*01∶01-HA_307–318(H6)_ tetramer. *In vitro* cultured PBMCs from a donor expressing HLA-DRB1*01∶01 were stained with increasing MHC class II tetramer concentrations (x-axis). The mean fluorescence intensity (MFI) obtained is shown on the y-axis. Circles illustrate data points of the MHC class II tetramer-labeled CD4+ T cell population (TMR+). Squares illustrate data points of MHC class II tetramer-negative CD4+ T cells (TMR-).

To identify the optimal staining conditions, we tested staining time and temperature. *In vitro* stimulated PBMC’s isolated from a HLA-DRB1*04∶01 positive donor with a cytokine response against the immunodominant Influenza HA_307–318_ peptide were stained with PE-conjugated HA_307–318(H6)_-HLA-DRB1*04∶01 tetramers for 60 min at 4°C, 25°C, 25°C (0.01% NaN_3_), or 37°C; or at 37°C for 5, 15, 30 or 60 min (Figure 2A). In accordance with other reports we found that staining was both dependent on time and temperature [32], [33], [34]. Staining the above PBMCs with a relevant (HA_307–318(H6)_-DRB1*04∶01) vs. an irrelevant (Hep C NS3_218–235(H6)_-DRB1*04∶01) tetramer showed baseline separation when staining for 15 min at 37°C, or at 25°C for 60 min. Furthermore, the binding of HLA class II tetramers to CD4+ T cells seemed to depend on active cellular processes, since a) staining in the presence of 0.01% azide reduced tetramer staining, and b) staining at 4°C, where membrane trafficking is abrogated, produced much less intense tetramer staining.

We subsequently selected 60 min at 37°C as routine staining conditions, and a titration from 0.1 nM to 250 nM of HA_307–318(H6)_-DRB1*01∶01 tetramers was performed to determine the optimal tetramer concentration (Figure 2B). This particular combination of tetramers and CD4+ T cells could be detected already at 1 nM and appeared saturated at 100 nM tetramer concentrations. In subsequent experiments we have chosen a tetramer concentration close to half-saturation (*in casu* 12.5 nM), as this separated tetramer positive CD4+ T cells and allowed the identification of subpopulations of CD4+ T cells with different avidities of tetramer-TcR interactions.

### Extending Tetramer Staining to Donors’ CD4+ T Cell Responses

The evaluation was subsequently extended to CD4+ T cell responses directed against three viral peptides identified in four different HLA-typed donors by ICS. A total of nine CD4+ T cell responses had been identified (Table 2). The three commonly recognized peptides included two derived from Influenza A, HA_307–318_ and MP_60–73_, both known to be CD4+ T cell epitopes [23], [24], and one CMV peptide, IE1_211–225_ (to be reported elsewhere). The four donors covered a total of five HLA-DR molecules, HLA-DRB1*01∶01, -DRB1*04∶01, -DRB1*15∶01, -DRB4*01∶03 and -DRB5*01∶01 (all, but HLA-DRB4*01∶03, were available to us). Examining the binding between the three peptides (with or without H_6_ tag) and the four available HLA class II molecules, showed medium to high affinity interactions with K_D_’s below 500 nM and in many cases below 50 nM (the exception being the missing interaction of the IE1_211–225_ peptide with HLA-DRB1*04∶01) (Table 1). Of these 11 productive peptide-HLA class II interactions, ten were successfully refolded as monomers and subsequently tetramerized (despite several attempts, HA_307–318(H6)_ failed to yield stable monomers with HLA-DRB1*15∶01). Thus, this strategy for production of HLA class II tetramers appears to be quite successful.

**Table 2:** Overview of donors, HLA class II DR alleles of individual donors, and cytokine (IFN-γ and TNF-α) responses measured with an intracellular cytokine secretion assay (ICS) against three viral CD4+ T cell epitopes.

					ICS		
Donor	HLA-type				Epitope		
	HLA-DR1		HLA-DR3,4,5		Influenza A HA_307–318_	Influenza A MP_60–73_	CMV IE1_211–225_
5	1*15∶01		5*01∶01		+	−	+
10	1*04∶01	1*15∶01	4*01∶03	5*01∶01	+	+	+
26	1*01∶01	1*04∶01	4*01∶03		+	+	−
40	1*01∶01	1*15∶01	5*01∶01		+	−	+

A positive response is indicated by +. A negative response is indicated by −.

**Figure 3:**
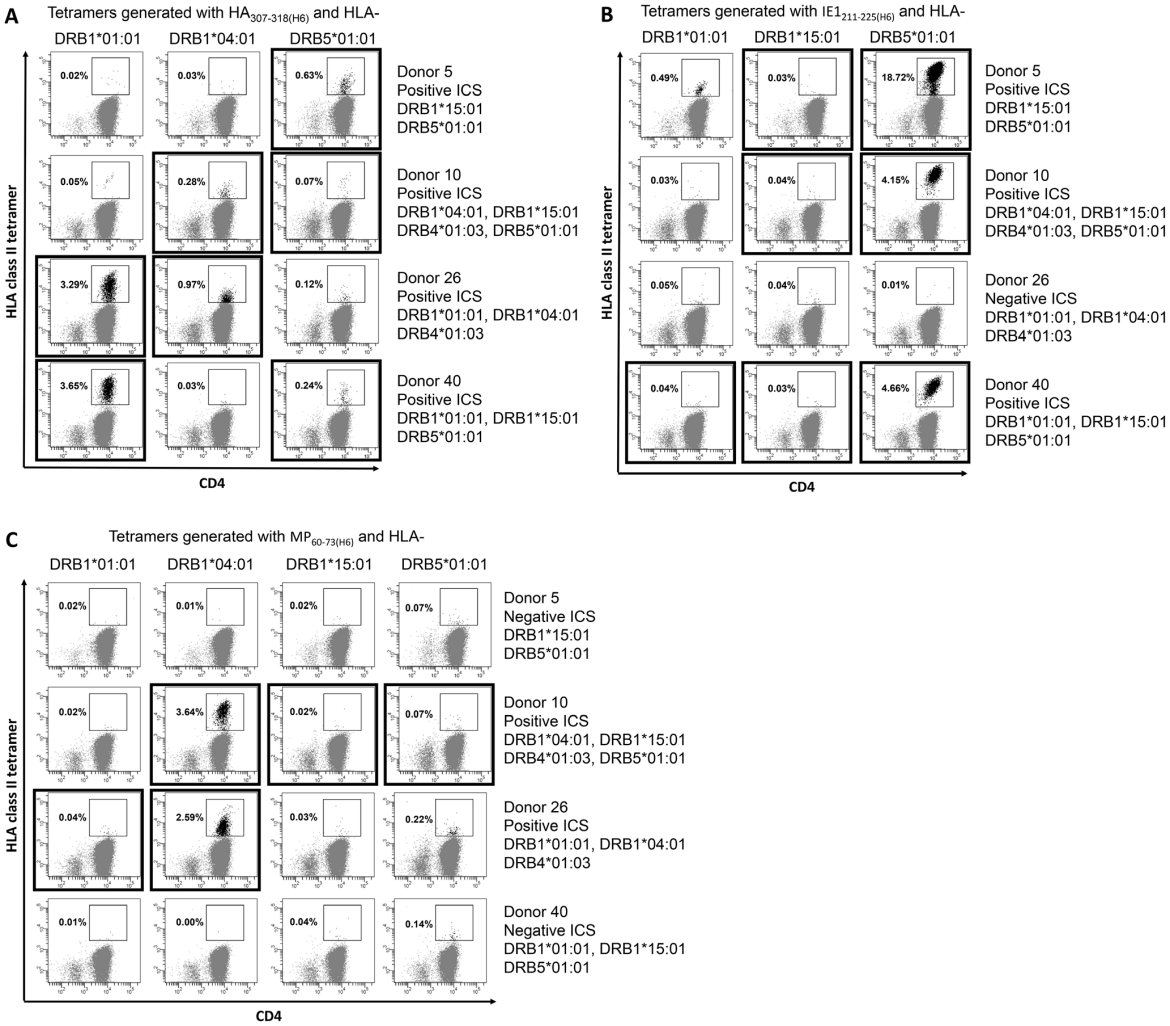
MHC class II tetramer stainings of Ag-specific CD4+ T cells. PBMCs from four donors were *in vitro* cultured with a mix of three different peptides (HA_307–318_, and IE1_211–225,_ and MP_60–73_) and analyzed with ten different tetramers. The tetramer used is indicated horizontally above each column of flow cytometry plots. Each row of these plots corresponds to one donor, where the HLA-DR-profile and cytokine response of this donor against the particular peptide is indicated. Plots representing tetramer labeling where the peptide/HLA class II components are deemed relevant (i.e. donor responded to the peptide and possessed the HLA class II molecule) are framed in bold. The frequencies (%) of CD4+ T cells that stain with the particular tetramers are indicated in each plot. A) A total of three different tetramers were made with HA_307–318(H6)_ and analyzed for the four donors. B) Three different tetramers were made with IE1_211–225(H6)_ and analyzed for the four donors. C) A total of four tetramers were made with MP_60–73(H6)_ and analyzed for the four donors.

To evaluate the tetramers, PBMCs from the four donors were *in vitro* stimulated with relevant peptide, expanded for 14 days, labeled with the appropriate T cell markers (i.e. anti-CD3 and anti-CD4 antibodies) and HLA class II tetramers, and then analyzed by flow cytometry. A priori, one would only expect tetramer staining of CD4+ T cells (and not of CD8+ T cells) in cases where the donor has responded to the peptide in question and the donor expresses the HLA class II allomorph that has been used to generate the tetramer. Figures 3A–C illustrates representative experiments, where the flow cytometry plots are framed in bold only when both of the above conditions are met.

Figure 3A shows the analysis of CD4+ T cell responses against the HA_307–318_ peptide, which was recognized by PBMCs from all four donors, and had been successfully tetramerized with three different HLA class II molecules. As an example, CD4+, and not CD8+, T cells from donor 5 were exclusively labeled with the HA_307–318(H6)_-HLA-DRB5*01∶01 tetramer; the only HA_307–318(H6)_-tetramer relevant to this donor. The two other HA_307–318(H6)_-tetramers (HA_307–318(H6)_-HLA-DRB1*01∶01 and HA_307–318(H6)_-HLA-DRB1*04∶01) did not label CD4+ T cells from donor 5. This was expected since these alleles are not present in this donor. Extending this analysis to the remaining three donors shows that one of two relevant tetramers strongly labeled CD4+ T cells from donor 10 (the other relevant tetramer labeled weakly), two out of two relevant tetramers strongly labeled CD4+ T cells from both donor 26 and 40. Note that the irrelevant tetramers labeled no or few CD4+ T cells (described in more detail below), and none of the tetramers stained CD8+ T cells.

Figure 3B shows the analysis of CD4+ T cell responses against the IE1_211–225_ peptide, which was recognized by PBMCs from three of the four donors, and had been successfully tetramerized with three different HLA molecules. PBMCs from donor 5 were labeled with one of the two relevant tetramers, the IE1_211–225(H6)_-HLA-DRB5*01∶01 tetramer, labeling about 20% of the CD4+ T cells of donor 5. Unexpectedly, the irrelevant IE1_211–225(H6)_-HLA-DRB1*01∶01 tetramer labeled about 0.5% of the CD4+ T cells obtained from this donor (examined in more detail below). Extending the analysis to the three remaining donors shows that one of two relevant tetramers strongly labeled CD4+ T cells from donor 10 (no labeling was observed with the other relevant tetramer). As expected, none of the tetramers labeled CD4+ T cells from donor 26, who did not have a CD4+ T cell response against IE1_211–225_ as judged by ICS analysis. One of three relevant tetramers strongly labeled CD4+ T cells from donor 40. Again, the irrelevant tetramers labeled no or few CD4+ T cells, and none of these tetramers stained CD8+ T cells.

Figure 3C shows the analysis of CD4+ T cell responses against the MP_60–73_ peptide, which was recognized by PBMCs from two of the four donors, and had been successfully tetramerized with four different HLA molecules. The relevant MP_60–73(H6)_-HLA-DRB1*04∶01 tetramer strongly labeled CD4+ T cells from the two responding donors, 10 and 26, but not from the non-responding donors, 5 and 40. Other tetramers might have been relevant for responding donors, 10 and 26, but they did not label CD4+ T cells from these donors. With one exception, the irrelevant tetramers labeled no or few CD4+ T cells (the exception was the irrelevant MP_60–73(H6)_-HLA-DRB5*01∶01 tetramers labeled about 0.3% of CD4+ T cells from donors 26 and 40). Again, none of the tetramers labeled CD8+ T cells.

In total, we examined nine different peptide-specific CD4+ T cell responses directed against three virus-derived peptides in four different donors. With access to four of the five HLA-DR molecules covered by the four donors, we could experimentally address 12 different peptide-HLA-DR combinations. Eleven of these combinations represented productive interactions (i.e. showing an affinity better than 500 nM), and ten of them were successfully used to generate tetramers. Labeling PBMCs from all four donors with all ten tetramers allowed us to evaluate staining efficiency and specificity in 40 experiments. Each tetramer labeled specific CD4+ T cells in one to three donors, whereas none of them labeled CD8+ T cells in any donor. A tetramer labeling was only considered relevant if a given donor both responded to the peptide in question and possessed the HLA-DR molecule in question. By this token, 19 of the 40 experiments involved relevant peptide-HLA-DR combinations. For each of the nine peptide-specific CD4+ T cell responses, the tetramers successfully identified from one to three relevant peptide-HLA-DR specific CD4+ T cell populations thereby validating the peptide-specificity and identifying the underlying HLA-DR restriction element. Tetramer staining was observed for a total of 12 relevant peptide-HLA-DR combinations and many of them involved high-intensity labeling of high-frequency CD4+ T cell populations. In contrast, low-intensity labeling of low-frequency populations was observed in only four cases. In at least two of these the apparently irrelevant labeling could be explained as cross-reactions at the level of HLA-DR (see below). Thus, successful tetramer labeling depended on the TcR, the HLA class II molecule, and the peptide, all being of the appropriate specificity. We conclude that our tetramer generation strategy is efficient, and that the resulting tetramers specifically label relevant T cell populations capturing the underlying peptide-specific, HLA class II-restricted CD4+ T cells.

### CD4+ T Cells May Cross-react Weakly with MHC Class II Tetramers Composed of Cognate Peptide and Allogeneic Class II Molecule

**Figure 4:**
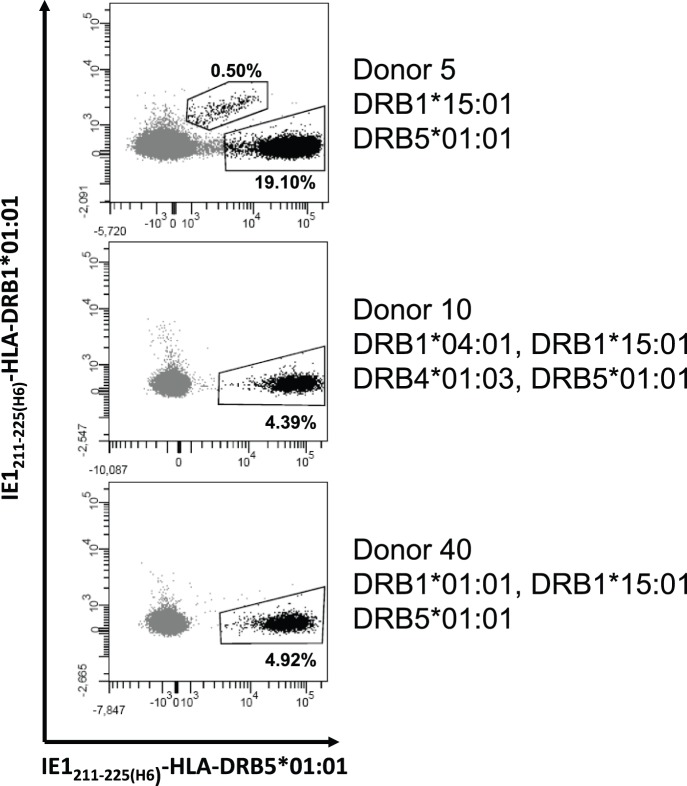
Double staining with different MHC class II tetramers. Flow cytometry plots of combined stainings with DRB1*01∶01-IE1_211–225(H6)_ tetramer and DRB5*01∶01-IE1_211–225(H6)_ tetramer of *in vitro* cultured PBMCs from three donors. Unexpectedly the DRB1*01∶01-IE1_211–225(H6)_ tetramer stained 0.5% CD4+ T cells in donor 5 although this donor did not express this allele. Double staining with DRB1*01∶01-IE1_211–225(H6)_ and DRB5*01∶01-IE1_211–225(H6)_ tetramer reveals that 0.5% of the much larger population recognizing IE1_211–225_ on DRB5*01∶01 cross-recognize the epitope on DRB1*01∶01.

Although of low intensity and frequency, the few examples of apparently irrelevant labeling were of considerable concern. Donor 5 exhibited the most pronounced labeling: 20% with the relevant IE1_211–225(H6)_ -HLA-DRB5*01∶01 tetramer and 0.5% with the irrelevant IE1_211–225(H6)_ -HLA-DRB1*01∶01 tetramer. This suggested to us that irrelevant labeling – at least in some cases – might be the result of a cross-reaction at the level of the HLA-DR component of the CD4+ T cell epitope, rather than the result of an unspecific interaction. To address this question experimentally, we generated the IE1_211–225(H6)_ -HLA-DRB5*01∶01 tetramer with an APC fluorochrome label and the IE1_211–225(H6)_ -HLA-DRB1*01∶01 tetramer with a PE fluorochrome label, and double-labeled PBMCs from donor 5 (Figure 4). This analysis demonstrated that about 19% of PBMCs from donor 5 were labeled exclusively with the IE1_211–225(H6)_ –HLA-DRB5*01∶01 tetramer, while 0.5% were labeled with both tetramers, and none were exclusively labeled by the IE1_211–225(H6)_ -HLA-DRB1*01∶01 tetramer. Thus, the apparent irrelevant labeling could in this case be fully accounted for by a cross-reaction at the level of the HLA-DR molecule, and the suspicion of unspecific tetramer labeling could be discarded. Note, that this particular HLA-DR-based cross-reaction was not observed in PBMCs from donors 10 and 40. A similar phenomenon of an apparently irrelevant labeling with HA_307–318_-HLA-DRB1*01∶01 tetramers being explained as a cross-reaction with the relevant HA_307–318_-HLA-DRB1*04∶01 tetramer was observed for PBMCs from donor 10 (data not shown). On a somewhat different note, these experiments illustrated another advantage of the double-labeling strategy where a pair of closely related, or even identical, peptide-HLA class II combinations can be tetramerized with different fluorochrome-labeled SAs and subsequently used to assist in a very precise delineation, and consequently a very accurate enumeration, of a T cell population of interest.

### Ex vivo HLA Class II Tetramer Staining and T Cell Sorting

**Figure 5:**
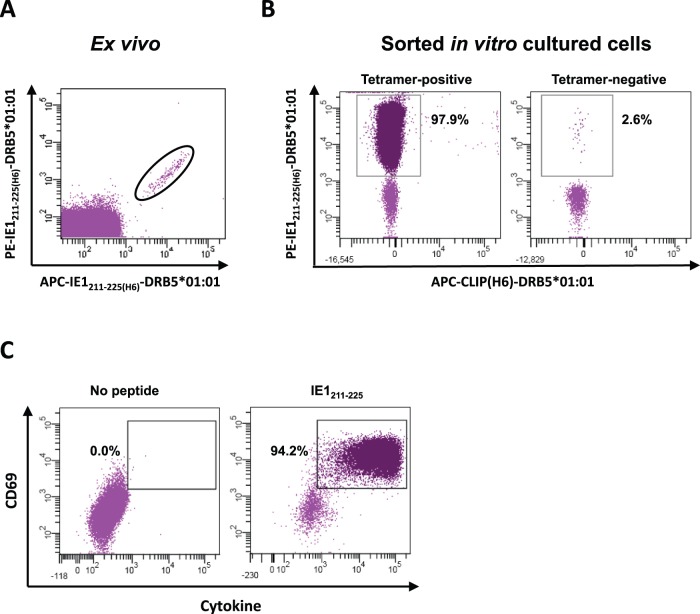
*Ex vivo* MHC class II tetramer staining and sorting. Two x 10^7^ PBMCs were incubated with PE- and APC-conjugated HLA-DRB5*01∶01-IE1_211–225(H6)_ tetramers for 2 h at 37**°**C. Approximately 1000 tetramer-positive cells were isolated on FACS-ARIAII and cultured for 14 days with irradiated autologous dendritic cells pulsed with IE1_211–225_. One thousand tetramer-negative, CD4+ T cells were isolated as control and cultured under identical conditions. Following *in vitro* culture, tetramer-positive cells expanded to approximately 6×10^5^ cells whereas tetramer-negative cells expanded to 10^4^ cells. A) Gated tetramer-positive CD4+ T cells (encircled) shown on FACS-ARIAII following *ex vivo* staining. B) Dot plots showing the percentage of cells that stain with tetramer following *in vitro* culture of tetramer-positive and tetramer-negative sorted cells. The cells were stained with PE-conjugated HLA-DRB5*01∶01-IE1_211–225(H6)_ (y-axis) and APC-conjugated HLA-DRB5*01∶01-CLIP(H6) (x-axis). Ninety-seven point nine % of *ex vivo* tetramer-positive sorted *in vitro* cultured cells stain with HLA-DRB5*01∶01-IE1_211–225(H6)_ (left dot plot) compared to 2.6% among *ex vivo* tetramer-negative *in vitro* cultured cells (right dot plot). No staining with irrelevant tetramer can be observed in either population. C) ICS of *in vitro* cultured tetramer-positive cells stimulated with IE1_211–225_–pulsed irradiated autologous dendritic cells. Ninety-four % produce cytokine (IFN-γ and TNF-α) and up regulate CD69.

The *ex vivo* frequency of antigen-specific CD4+ T cell responses is generally considered to be very low and difficult to detect by tetramer staining. To investigate whether antigen-specific CD4+ T cells can be *ex vivo* stained and purified, PBMCs from donor 40 were thawed, rested for 2 h at 37**°**C, and then double-labeled with PE- and APC-conjugated IE1_211–225(H6)_-HLA-DRB5*01∶01 tetramers at 37**°**C for 2 h. Following staining with anti-CD3 and anti-CD4 antibodies, the PBMCs were then sorted on a FACS Aria II yielding approximately 1000 double stained CD4+ T cells out of an input of 2×10^7^ sorted PBMCs, or a frequency of about 50 per million. The cells were plated in a 96-well round bottom microtiter plate. As a control, 1000 tetramer-negative CD4+ T cells from the same sort were also plated. Irradiated autologous dendritic cells were pulsed with IE1_211–225_ and added at a ratio of 1∶3 (CD4+ T cells/dendritic cells) to each wells. The cells were expanded with cytokines (see materials and methods) for 14 days and harvested. A total of 6×10^5^ cells (a 600-fold expansion) were obtained from the tetramer stained CD4+ T cells; 97.9% of these were IE1_211–225(H6)_-HLA-DRB5*01∶01 tetramer-positive and more than 94% responded with cytokine secretion following exposure to autologous dendritic cells pulsed with IE1_211–225_. In contrast, only 10^4^ cells were obtained from the expanded control cells and only 2.6% of these were tetramer-positive (Figure 5). There were too few cells to perform ICS. Thus, the HLA class II tetramers can be used to stain and purify very rare antigen-specific CD4+ T cells.

### Tetramers Incorporating H_6_-tagged Peptides Largely Stain the Same CD4+ T Cells as those Incorporating Non-tagged Peptides

**Figure 6:**
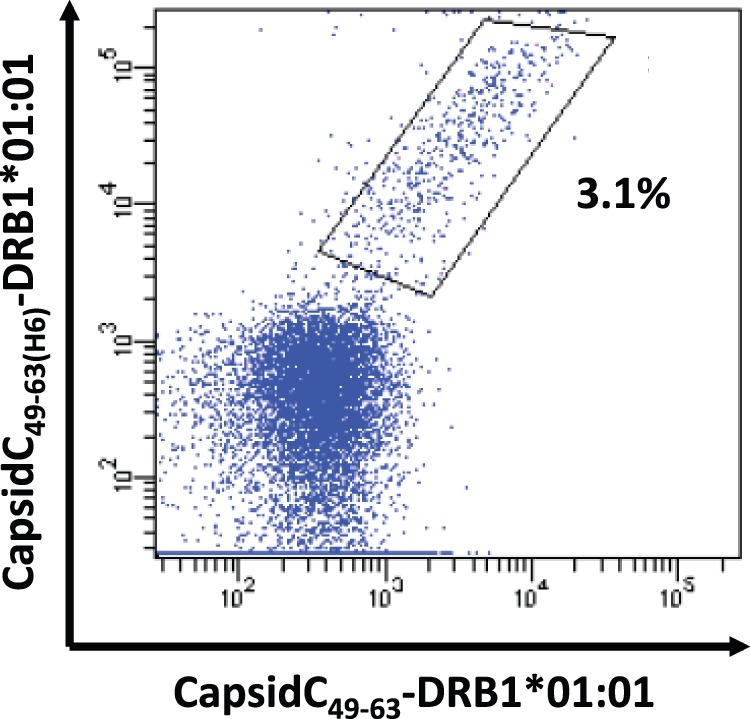
Head to head comparison of MHC class II tetramers generated with H_6_-tagged peptides versus non-H_6_-tagged peptides. PBMCs *in vitro* cultured with a yellow fever epitope were stained with two tetramers carrying the same peptide-HLA class II specificity (Yellow fever virus CapsidC_49–63_ restricted to HLA-DRB1*01∶01). Monomeric peptide-HLA class II complexes generated with H_6_-tagged peptide were tetramerized with PE-conjugated SA (y-axis). Monomeric peptide-HLA class II complexes generated with non-H_6_-tagged peptide were tetramerized with APC-conjugated SA (x-axis). All CD4+ T cells stained with non-H_6_-tagged tetramer are also stained with the H_6_-tagged tetramer.

Having established H_6_-tagged peptides as a useful mean to purify peptide-HLA class II monomers for subsequent tetramers generation, we returned to the issue of whether the C-terminal addition of a tag could alter the specificity of the resulting tetramers. Initially, we demonstrated that the tag did not grossly alter the specificity or affinity of peptide binding to HLA class II molecules. However, this did not rule out that the tag could interfere with T cell recognition. To address this directly, we generated two tetramers carrying the same peptide-HLA class II specificity (a yellow fever virus capsid peptide-specific, HLA-DRB1*01∶01-restricted specificity to be reported elsewhere); one labeled with APC where a standard approach with non-H_6_-tagged peptide had been used to generate monomers, and one labeled with PE where the H_6_-tagging approach had been used. Double staining of relevant CD4+ T cells demonstrated that all cells that were stained with the non-tagged tetramer were also stained with the tagged tetramer (Figure 6). Reassuringly, the CD4+ T cells did not seem to distinguish between the two tetramers.

## Discussion

CD4+ T cells are specific for peptide antigens (epitopes) presented in association with MHC class II molecules [1]. Accurate determination of this specificity requires that both the epitope and the restricting MHC class II element can be determined and validated. This information is essential for our understanding of CD4+ T cell mediated immunity and for possible applications hereof. Unfortunately, the determination of these two highly variable and polymorphic components of T cell specificity is complicated by degeneracy, or promiscuity, of recognition both at the level of the MHC class II and TcR [35]. This compromises the interpretation of many, in particular functional, assays in current use (e.g. ELISpot and ICS). In particular, the identification of MHC class II restricting elements is a strenuous experimental challenge, which includes many different elements such as blocking experiments with anti-MHC class II antibodies, analysis of CD4+ T cell responses of cohorts of HLA-typed donors, biochemical analysis of peptide binding to MHC class II molecules etc. Indeed, a recent paper from Sette et al. is devoted to the establishment of a panel of single class II transfected cell lines, which promises to enable a comprehensive approach to the determination of MHC class II restriction elements involved in a given CD4+ T cell response [35]. MHC class II tetramers aim to solve the combined task of determining the peptide(s) and MHC class II molecule(s) involved in generating the TcR ligand(s) of a given CD4+ T cell response. Due to practical limitations, however, access to MHC class II tetramers has been severely restricted. Whereas the availability of easy and efficient methods to produce the needed MHC class I molecules has contributed to the success of MHC class I tetramers, the lack of versatile and efficient MHC class II production methods can in part explain the limited access to MHC class II tetramers.

We have recently described a strategy to produce recombinant MHC class II molecules by expression of transmembrane-truncated versions of isolated α and *in vivo* biotinylated β chain molecules in *E. coli* [19]. Briefly, these chains are extracted individually into 8 M Urea in the absence of any reducing agents, then purified and stored at −80°C. These isolated MHC class II α and β chain molecules can be thawed and recombined at the time of refolding, diluted into an aqueous refolding buffer with the appropriate peptide of choice, and incubated at 18°C for 48 h to allow for efficient complex formation. We have previously demonstrated that recombinant pre-oxidized MHC or MHC-like molecules refold rapidly and very efficiently [18], [19], [36]. These pre-oxidized HLA class II α and β chains are active, allow the generation of predetermined homogenous peptide-HLA class II complexes, and support peptide-class II binding assays [19]. A clear advantage of using isolated chains is that they support a modular approach where combining pairs of a limited number of α and β chains can generate a very large number of different HLA class II molecules; something that is particularly useful for HLA-DQ and -DP molecules where both the α and β chains are polymorphic. Furthermore, these molecules are empty and capable of binding any suitable (i.e. binding) peptide allowing the generation of an extremely large number of different peptide-HLA class II complexes. In contrast, alternative approaches to generate recombinant class II molecules have in some cases called for the α and β chains being expressed as a pair requiring a unique production of each pair of interest [10], [37], [38], [39], and in other cases called for the target peptide being expressed as part of a fusion construct requiring a unique preparation for each peptide-HLA class II complex of interest [14].

Although we have successfully generated a large number of functional HLA class II molecules from all three HLA isotypes (HLA-DR, -DQ and DP), we have generally failed to use these reagents to generate class II tetramers of a quality that allows efficient staining of CD4+ T cells (data not shown). This is in stark contrast to the high quality of class I tetramers that can be obtained with pre-oxidized class I molecules [21]. One crucial difference between pre-oxidized recombinant class I and II molecules relates to the efficiency of refolding when renatured in aqueous buffer in the presence of peptide. Class I molecules can refold with almost 100% efficiency; in fact, such a preparation can be used for tetramer formation without further purification [21]. In contrast, class II molecules refold with less efficiency [19] leading to the generation of a mixture of correctly and incorrectly folded molecules making purification of correctly folded molecules, a prerequisite for tetramer formation. This is not trivial as even incorrectly folded α and β chains can pair due to the leucine zipper-driven assembly strategy. We have previously used N-terminally tagged peptides to purify correctly folded HLA class II monomers [19]. Here, we have changed this to a C-terminal tagging principle. Albeit this is a quite minor modification, this is a far more versatile and less expensive approach from a peptide synthesis perspective, since a large batch of peptide resin can be pre-prepared and used as a convenient starting point for subsequent synthesis of affinity-tagged peptides. We have applied this principle to monomer purification and demonstrate that it readily supports subsequent HLA class II tetramer generation thus extending our class II production and assay technology to also support a versatile class II tetramer technology. In agreement, others have noticed that classical purification strategies like gel size filtration chromatography fail to generate monomers of sufficient quality for tetramer formation and shown that N-terminally extended tagged peptides enable the purification of correctly folded HLA class II monomers and subsequent tetramer formation [33]. Also in agreement with previously published studies [32], [33], [34], we show that class II tetramer staining is facilitated at physiological temperatures and prolonged exposure times, and involves intact metabolism.

In contrast to MHC class I, the open structure of the peptide-binding cleft of MHC class II molecules should in general be amenable to strategies involving tagging of the peptide. However the addition of amino acids unrelated to the original antigenic peptide sequence could be a possible limitation of the approach due to interference with MHC class II binding and/or with the subsequent TcR recognition event. Our data on the MHC class II binding affinity of tagged vs. non-tagged peptides, and on CD4+ T cell staining with tagged vs. non-tagged peptides, demonstrated that MHC class II and/or TcR interference is not a general problem. Whether a tag should interfere with MHC class II interaction itself can easily be examined in peptide-binding assays, but whether it may interfere with T cell and/or TcR interaction is much more difficult to determine. There are examples of both N- and C-terminal flanking manipulations affecting T cell repertoire selection [40], [41]. Although one should always be aware that a tagged peptide might miss a specific clonal T cell reactivity, our data argue that a polyclonal response in general will be detectable with C-terminally tagged peptide-class II tetramers. Although our data suggest that a C-terminal tagging principle may not be a general problem, one should not rule out that peptide tagging could be a significant problem for specific peptide-MHC-TcR interactions.

Here, we performed a comprehensive analysis of a few CD4+ T cell responses in a few donors. This illustrates the advantages of HLA class II tetramers and raises a caveat. The complicated multiple-restricted nature of CD4+ T cell responses is a relevant issue: only one of nine CD4+ responses was restricted solely by one of the HLA-DR restriction elements; the remaining eight responses were restricted by two to three different HLA-DR restriction elements available to a given donor. As a unique advantage of HLA class II tetramers, these could decisively determine and enumerate which of several possible restriction elements were in use in each donor. The promiscuous nature of CD4+ T cell recognition is also significant: in several cases, donor CD4+ T cells could not only be labeled with an appropriate peptide-HLA class II tetramer (i.e. where the donor possessed the HLA class II used to generate the tetramer), but, albeit less intense and less frequent, also by inappropriate tetramers (i.e. where the donor did not possess the HLA class II). Subsequent double staining experiments revealed that all inappropriately labeled T cells could also be labeled with the appropriate tetramer, i.e. that the inappropriate labeling could be explained fully as an HLA class II cross-reaction by the promiscuous TcR, rather than by a non-specific interaction. This must be taken into account as a caveat in any attempt to determine the restriction of a T cell response in the absence of HLA typing; a caveat that is equally relevant in strategies using panels of tetramers or panels of single HLA class II transfected cells to determine restriction.

We would like to comment on the findings of others who have suggested that peptides with multiple class II binding registers lead to heterogeneous peptide-class II monomers and that this should be the most important factor limiting the ability to generate class II tetramers [42]. At this point, we have applied the strategy described here to generate 66 different HLA-DR tetramers covering 11 different HLA-DR molecules (B1*01∶01, B1*03∶01, B1*04∶01, B1*07∶01, B1*08∶01, B1*13∶01, B1*13∶02, B1*15∶01, B3*01∶01, B3*03∶01, B5*01∶01 (as of July 2013, our laboratory has produced 30 different recombinant HLA-DR molecules)). We have used these 66 class II tetramers to analyze a large number of anti-viral CD4+ T cell responses and thereby identified 37 different CD4+ T cell epitopes. In others words, we have a success rate of 37/66 or 56%. The remaining 44% may in many cases not represent failures, but merely instances where the TcRs corresponding to the chosen tetramers were not present in these donors. These data would suggest that multiple registers is not as pronounced a problem as previously suggested.

In conclusion, our data suggest that individually prepared recombinant class II α and β chains in conjunction with C-terminally tagged peptides represent a general principle applicable for efficient, versatile MHC class II tetramer production.
