# Correction: MHC Class II Tetramers Made from Isolated Recombinant α and β Chains Refolded with Affinity-Tagged Peptides

**DOI:** 10.1371/annotation/97cabee9-2a2a-4bd6-a417-a2075e56a8d4

**Published:** 2013-09-24

**Authors:** Peter Braendstrup, Sune Justesen, Thomas Østerbye, Lise Lotte Bruun Nielsen, Roberto Mallone, Lars Vindeløv, Anette Stryhn, Søren Buus

This article has been republished to correct errors in Figure 3. 

