# Correction: MHC Class II Tetramers Made from Isolated Recombinant α and β Chains Refolded with Affinity-Tagged Peptides

**DOI:** 10.1371/annotation/9375e7cb-92f1-4a83-adaf-c11f284ceee0

**Published:** 2013-12-10

**Authors:** Peter Braendstrup, Sune Justesen, Thomas Østerbye, Lise Lotte Bruun Nielsen, Roberto Mallone, Lars Vindeløv, Anette Stryhn, Søren Buus

There is an error in the legend of Figure 1. The correct legend is:

Figure 1. Purification of H6-tagged MHC class II complexes. A) Preparative purification of DRB1*04:01 refolded with the Influenza A peptide HA307–318(H6) (YKYVKQNTLKLATHHHHHH) on IMAC column. A shallow gradient up to 50mM Imidazole in PBS (20% buffer B) eluted peak 1 containing alpha and beta chains without associated peptides, whereas peak 2, containing mature MHC II complexes, eluted by raising the concentration to 250mM imidazole in PBS (100% buffer B). B) SDS-PAGE analysis of eluted fractions, the alpha chain travels as the upper band. C) Functional LOCI assay on load, run through, and peak fractions. Notice the absence of peptide-loaded MHC class II molecules in the run through (RT) and in peak 1. D) Assessing the level of biotinylation of a DRB1*11:01 chain by incubation with increasing amounts of avidin followed by SDS-PAGE analysis.

